# Oncogenic addiction to high 26S proteasome level

**DOI:** 10.1038/s41419-018-0806-4

**Published:** 2018-07-10

**Authors:** Peter Tsvetkov, Julia Adler, Nadav Myers, Assaf Biran, Nina Reuven, Yosef Shaul

**Affiliations:** 1 Department of Molecular Genetics, Weizmann Institute of Science, 76100 Rehovot, Israel; 2 Broad Institute of MIT and Harvard, 415 Main St., Cambridge, MA 02142 USA

## Abstract

Proteasomes are large intracellular complexes responsible for the degradation of cellular proteins. The altered protein homeostasis of cancer cells results in increased dependency on proteasome function. The cellular proteasome composition comprises the 20S catalytic complex that is frequently capped with the 19S regulatory particle in forming the 26S proteasome. Proteasome inhibitors target the catalytic barrel (20S) and thus this inhibition does not allow the deconvolution of the distinct roles of 20S versus 26S proteasomes in cancer progression. We examined the degree of dependency of cancer cells specifically to the level of the 26S proteasome complex. Oncogenic transformation of human and mouse immortalized cells with mutant Ras induced a strong posttranscriptional increase of the 26S proteasome subunits, giving rise to high 26S complex levels. Depletion of a single subunit of the 19S RP was sufficient to reduce the 26S proteasome level and lower the cellular 26S/20S ratio. Under this condition the viability of the Ras-transformed MCF10A cells was severely compromised. This observation led us to hypothesize that cancer cell survival is dependent on maximal utilization of its 26S proteasomes. We validated this possibility in a large number of cancer cell lines and found that partial reduction of the 26S proteasome level impairs viability in all cancer cells examined and was not correlated with cell doubling time or reduction efficiency. Interstingly, normal human fibroblasts are refractory to the same type of 26S proteasome reduction. The suppression of 26S proteasomes in cancer cells activated the UPR and caspase-3 and cells stained positive with Annexin V. In addition, suppression of the 26S proteasome resulted in cellular proteasome redistribution, cytoplasm shrinkage, and nuclear deformation, the hallmarks of apoptosis. The observed tumor cell-specific addiction to the 26S proteasome levels sets the stage for future strategies in exploiting this dependency in cancer therapy.

## Introduction

Proteasomal protein degradation is crucial in maintaining cellular integrity, regulating cell cycle, proliferation, and cell death^1^. Proteasomal degradation of proteins is mediated by two distinct proteasome complexes—the 26S and the 20S proteasomes. The 26S proteasome consists of the 20S catalytic domain assembled with either one or two 19S regulatory particles (RP)^2,3^. In the well-characterized ubiquitin-proteasome system (UPS) a protein substrate is targeted to the 26S proteasome following conjugation of a poly-ubiquitin chain^1,4^. The polyubiquitinated substrate is then recognized by specific subunits of the 19S RP of the 26S proteasome where it is deubiquitinated, unfolded by the ATPases and translocated into the 20S catalytic chamber for degradation^2,5,6^ Over the years an alternative, ubiquitin-independent proteasome degradation pathway has been described whereby intrinsically disordered proteins (IDPs) such as p53, c-Fos, and BimEL^7–9^ (and others as reviewed in refs. ^10,11^) are degraded by the 20S proteasome in a process that does not involve active ubiquitin tagging^12,13^. Thus, there are at least two distinct proteasome protein degradation pathways, each regulated by the distinct 26S and 20S proteasome complexes.

The UPS as a regulator of cell death has been a tempting target for drug development for many pathologies, including cancer^14–16^. Various tumors have been shown to express high levels of proteasome subunits and higher proteasome activity^17,18^. A number of studies suggest that cancer cells exhibit high sensitivity to proteasome inhibition^19^. Proteasome inhibition is a strategy utilized in treating lymphoid malignancies, particularly multiple myeloma where the proteasome inhibitor bortezomib (VELCADE and PS-341) is in use for therapy^20^. Proteasome inhibitors were also shown to be effective in various screens of solid and hematologic tumors^19,21,22^. Proteasome inhibitors such as bortezomib, MG132, and carfilzomib inhibit the catalytic activity within the 20S proteasome particle that is essentially responsible for the activity of both the 20S and the 26S proteasomes^23^. Therefore, these drugs cannot be utilized to characterize or distinguish between any unique functions that either the 20S or the 26S proteasome complex play in the cell.

The cellular levels and 26S/20S proteasome complex ratio is both dynamic and regulated. Recent studies showed that a common mechanism of resistance to proteasome inhibitors involves the reduction in the cellular 26S/20S proteasome complex ratio^24–26^. These findings highlight the notion that the ratio of proteasome complexes in the cell is a regulated and crucial process. Further findings demonstrate the functional alteration of the 26S/20S proteasome complex ratio in the context of cell cycle progression^27^, neuronal function^28,29^, metabolic regulation^30–33^, and aging^34–36^.

During the process of transformation there is increased dependency on proteasome function as part of global increased burden on the protein homeostasis machinery^37^. Genetic screen analysis in several models including Ras transformation and triple negative breast cancer cells revealed a strong dependency on the proteasome function^38–40^. These findings motivated us to explore the alteration in the 26S–20S proteasome complex levels and ratio in oncogenic transformation. Utilizing an isogenic cell line model, we challenged the prediction that cancer cells are specifically vulnerable to the reduction of the 26S proteasome complex. The result of our study led us to the discovery that highly transformed cells exhibit increased levels of the 26S proteasome complexes and are extremely sensitive to their specific suppression.

## Results

### 26S proteasome levels increase following H-Ras V12 transformation

**Fig. 1:**
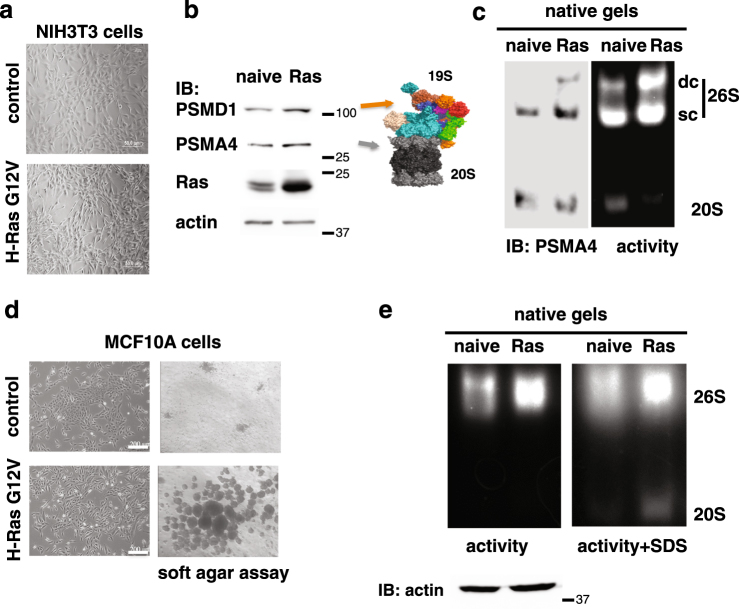
The H-Ras G12V-transformed NIH3T3 and MCF10A cells contain high levels of 26S proteasome. **a** NIH3T3 cells were transduced with retroviruses carrying H-Ras G12V gene, and visualized by microscopy. The cells displayed a transformed phenotype with a spindle-shaped and highly refractile morphology. **b** Expression of the proteasomal subunits PSMA4, a 20S component, and PSMD1, a 19S component, was tested by immunoblot (IB). **c** 26S and 20S proteasomal complex activity and levels were analyzed by native gel electrophoresis in both naive NIH3T3 and Ras-transformed cells. Equal amount of total protein, determined by Bradford assay, was loaded onto native gels (actin loading control is represented). The 26S complex is either double capped, namely each of both ends of the 20S proteasome is occupied by a 19S complex (DC-26S) or single capped (SC-26S). **d** MCF10A were transduced with retroviruses carrying pBabe H-Ras G12V gene, visualized by microscopy and subjected to soft agar assay. Transformation was evidenced by a fibroblast-like appearance and a dispersed cell distribution in monolayer culture (left) and by anchorage-independent growth in soft agar (right). **e** 26S and 20S proteasomal complex activity and levels were analyzed by native gel electrophoresis in both naive MCF10A and Ras-transformed cells. In the right panel, for the visualization of the 20S complex, the proteasomal activity was enhanced by the addition of 0.02% SDS to the activity reaction

Little is known on the alteration of the different proteasome complexes (26S and 20S) during the process of cancer transformation. Initially, we set out to examine the specific levels of the 26S proteasome upon cell transformation. To this end, we overexpressed the mutant H-Ras V12 (G12V) to transform the immortalized NIH3T3 cells (Fig. 1a). The level of the PSMA4, a component of the 20S proteasome, and PSMD1, a component of the 19S RC, were partially increased after transformation (Fig. 1b). However, in the transformed cells the level of the assembled 26S proteasome, in particular the double-capped complex, was markedly increased (Fig. 1c).

Next, we H-Ras transformed the human immortalized MCF10A cell line. The transformed cells proliferated not significantly higher than the naive cells (Supplementary Figure S1), but unlike the naive cells they formed colonies in soft agar gel, suggesting they acquired anchorage-independent growth, a hallmark of transformation (Fig. 1d). In the transformed cells the level of the proteasomes was higher than in the naive (immortalized) cells (Fig. 1e). These data suggest that H-Ras V12 transformation of mouse and human cells is associated with an increase of functional proteasome complexes.

### Proteasome subunits accumulate in H-Ras G12V-transformed MCF10A cells

**Fig. 2:**
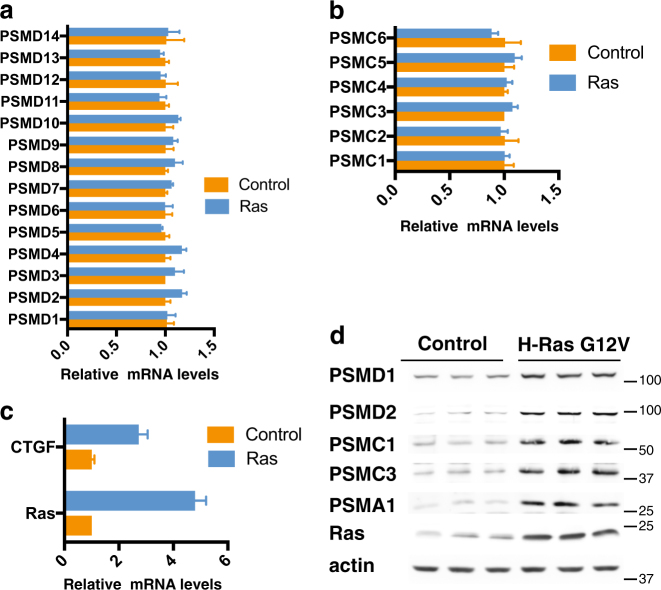
H-Ras G12V-transformed MCF10A cells show increased protein level of the components of the 19S subunits. MCF10A were transduced with retroviruses carrying H-Ras G12V gene. mRNA levels of all PSMD (**a**) and PSMC (**b**) proteasomal subunits, as well as of Ras and CTGF genes (**c**), were measured by qPCR. Protein levels of selected proteasomal subunits were tested in triplicate by immunoblot (**d**)

The H-Ras V12-mediated increase in 26S proteasome complex levels could be due to either a transcriptional or posttranscriptional event. No significant differences in the 19S PSMD (Fig. 2a), PSMC (Fig. 2b), and 20S PSMA and PSMB (Supplementary Figure S2) subunits mRNA levels were observed between the immortalized and transformed cells. As a positive control, we quantified the mRNA levels of CTGF, a hallmark of transformation, which was upregulated in H-Ras V12-transformed MCF10A cells (Fig. 2c). However, immunoblot analysis revealed higher protein levels of the selected members of the 19S RC (PSMDs and PSMCs) and the 20S (PSMA1) subunits in the MCF10A-transformed cells (Fig. 2d triplicates). Interestingly, the obtained fold of increase was similar to that of H-Ras V12 level, despite the fact that the Ras mRNA level was fivefolds higher in the transformed cells. These data suggest that upon transformation, cells accumulate much higher levels of the 26S proteasome by improving the translation and/or stability of the different proteasome subunits.

### H-Ras G12V-transformed MCF10A are more sensitive to 26S proteasome complex depletion

**Fig. 3:**
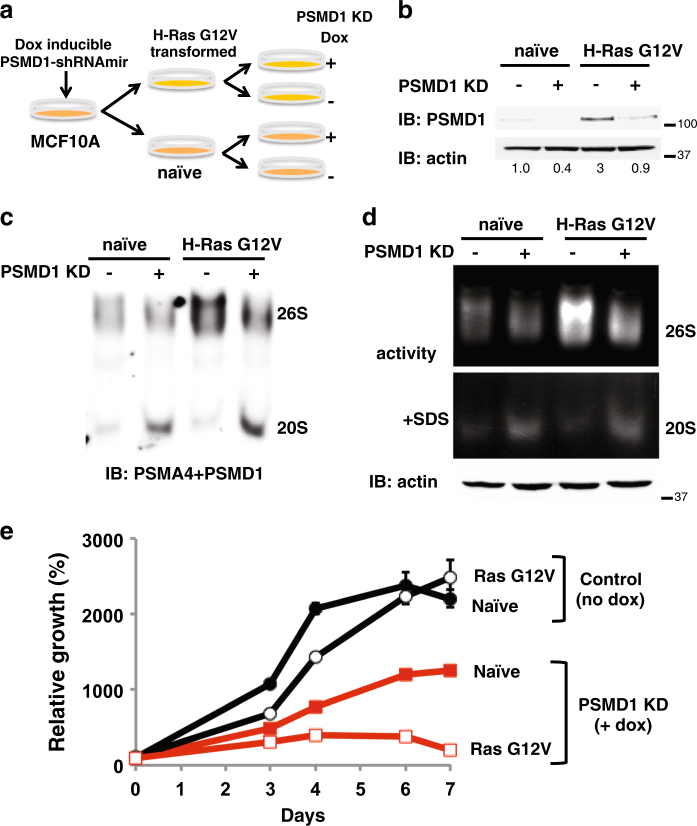
H-Ras G12V-transformed MCF10A are addicted to high 26S proteasome levels. **a** Schematic description of the experimental strategy. MCF10A stably expressing doxycycline-inducible PSMD1 shRNA were further subjected to transformation with H-Ras G12V. PSMD1 KD was induced by the addition of 1 μg/ml doxycycline for 72 h. **b** Protein content in the MCF10A naive and H-Ras G12V-transformed cells, in the presence or absence of PSMD1 shRNA induction, was analyzed by immunoblot. Quantification of the amount PSMD1, normalized to the amount of actin, is presented. **c** The 26S and 20S proteasomal complex levels were analyzed by native gel electrophoresis as described in Fig. 1c. **d** The levels of the 26S and 20S proteasome activity were determined as in Fig. 1c. Equal amounts of total protein, determined by Bradford assay, were loaded onto native gels (actin loading control is represented). **e** Cell proliferation rate was analyzed using the XTT assay. Naive or Ras-transformed MCF10A cells harboring a doxycycline-inducible PSMD1 shRNA were either doxycycline treated to induce PSMD1 shRNA expression or left untreated, and cell proliferation was followed daily. XTT at the seeding day was taken as 100% in measuring relative cell growth

Given the high 26S proteasome levels in H-Ras V12-transformed cell lines, we set out to explore the degree of dependency of the transformed state on high 26S proteasome levels. To do so, we established an isogenic model of H-Ras V12 transformation, where specific reduction of 26S proteasomes is achieved by transient overexpression of an shRNA targeting the 19S subunit PSMD1. We first transduced MCF10A cells with a lentivector (LV) expressing a doxycycline (dox)-inducible shRNA targeting the 19S subunit PSMD1 (Fig. 3a). Cells were then transformed by overexpression of the oncogenic H-Ras G12V and as expected, a higher level of PSMD1 was obtained (Fig. 3b). Upon dox treatment, the PSMD1 level was markedly reduced. PSMD1 depletion also resulted in decreased levels (Fig. 3c) and activity (Fig. 3d) of the 26S proteasome complex. We will refer to this process as 26S depletion.

The viability of the H-Ras V12-transformed but not the naive MCF10A cells was strongly compromised upon 26S depletion (Fig. 3e). This finding was unexpected since the 26S proteasome is higher in the transformed cells (Fig. 3c, d). Thus, the utilization of the maximal high level of 26S proteasomes in the H-Ras V12-transformed cells is crucial for their survival hinting toward an oncogenic addiction to the elevated 26S proteasome levels.

### Increased dependency on 26S proteasome levels in a triple negative breast cancer cell line

**Fig. 4:**
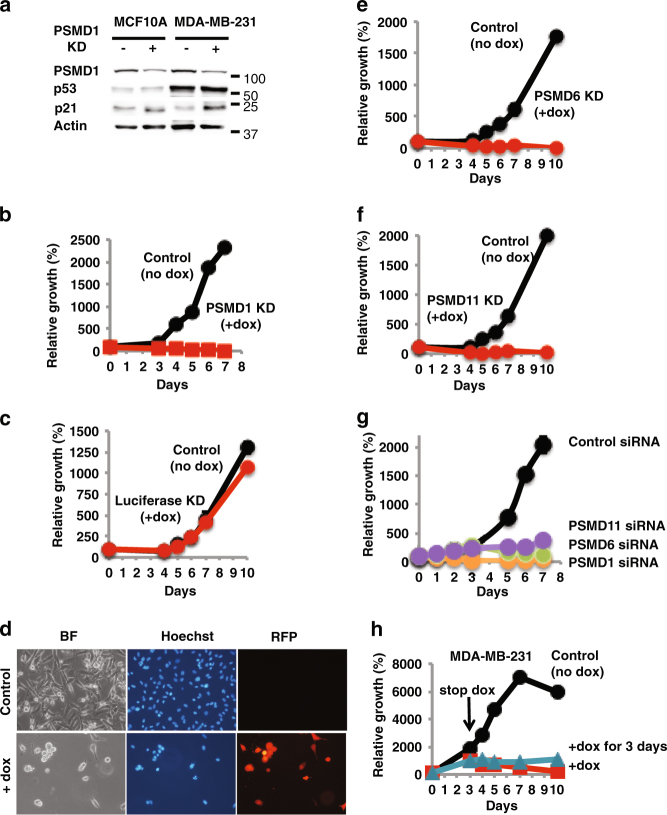
A triple negative breast cancer cell line is addicted to high 26S proteasome levels. **a** MCF10A and MDA-MB-231 cells harboring a doxycycline-inducible PSMD1 shRNA were either doxycycline treated to induce PSMD1 shRNA expression or left untreated. The levels of p53, p21, and PSMD1 were analyzed by immunoblot. **b** Growth of MDA-MB-231 cells expressing doxycycline-inducible PSMD1 shRNA was analyzed using the XTT assay. **c** Growth of MDA-MB-231 cells expressing doxycycline-inducible shRNA designed against the luciferase gene was analyzed as in **b**, ruling out non-specific doxycycline effects. **d** Visualization of the 26S-depleted MDA-MB-231 cells 4 days after PSMD1 shRNA induction. Expression of RFP as a marker for shRNA expression, and nuclear staining by DAPI are shown. Growth of MDA-MB-231 cells expressing doxycycline-inducible PSMD6 shRNA (**e**) and PSMD11 shRNA (**f**) was analyzed as in **b**. **g** Effect of synthetic siRNA designed against PSMD1, 6 or 11 or the luciferase gene on growth of MDA-MB-231 cells. Cells were transfected with the above siRNAs, and, after 24 h were replated for the XTT assay. **h** Irreversible inhibition of cell proliferation and induction of cell killing in MDA-MB-231 cells by PSMD1 shRNA expression. Cells were induced to express PSMD1 shRNA for 3 days by doxycycline, and then washed and cultured in fresh medium without or with doxycycline for an additional 7 days. Cell proliferation was analyzed as in 4**b**

We next examined MDA-MB-231, a triple negative breast tumor cell line, utilizing the same PSMD1 depletion strategy. A reduction in PSMD1 levels was obtained in these cells and the control MCF10A cells (Fig. 4a). As expected, the level of p53 is high in MDA-MB-231 cells^41^ but was not affected by 26S depletion. The level of p21 was increased in response to 26S depletion, which may result in cell cycle arrest. Since the MDA-MB-231 cells have a mutant p53, the obtained effect on cell viability is p53 independent. The reduction at the level of PSMD1 was sufficient to completely inhibit MDA-MB-231 cell growth (Fig. 4b), whereas only a minor effect was observed on the MCF10A cells (Fig. 3e). When shRNA designed against the Luciferase gene was induced by doxycycline, cell growth was not affected (Fig. 4c), ruling out non-specific doxycycline effects. Four days after 26S proteasome depletion only a few viable cells were observed exhibiting condensed nuclei (Fig. 4d). Similar results were obtained with 26S depletion achieved by shRNAs targeting two other 19S complex subunits, namely PSMD6 (Fig. 4e) and PSMD11 (Fig. 4f) and with synthetic siRNA designed against each of these three subunits (Fig. 4g). Both shRNA and siRNA designed against each of these three subunits were effective in knocking down the respective PSMD mRNA levels (Supplementary Figure S4). Furthermore, the detrimental effect of 26S proteasome depletion in MDA-MB-231 cells was irreversible. PSMD1 shRNA expression for only 3 days was sufficient to induce irreversible inhibition of cell proliferation and induction of cell killing (Fig. 4h). These data suggest that the TNBC cells might be particularly addicted to the 26S proteasome level.

### Aggressive and drug-resistant tumor cell lines are more susceptible to 26S depletion

**Fig. 5:**
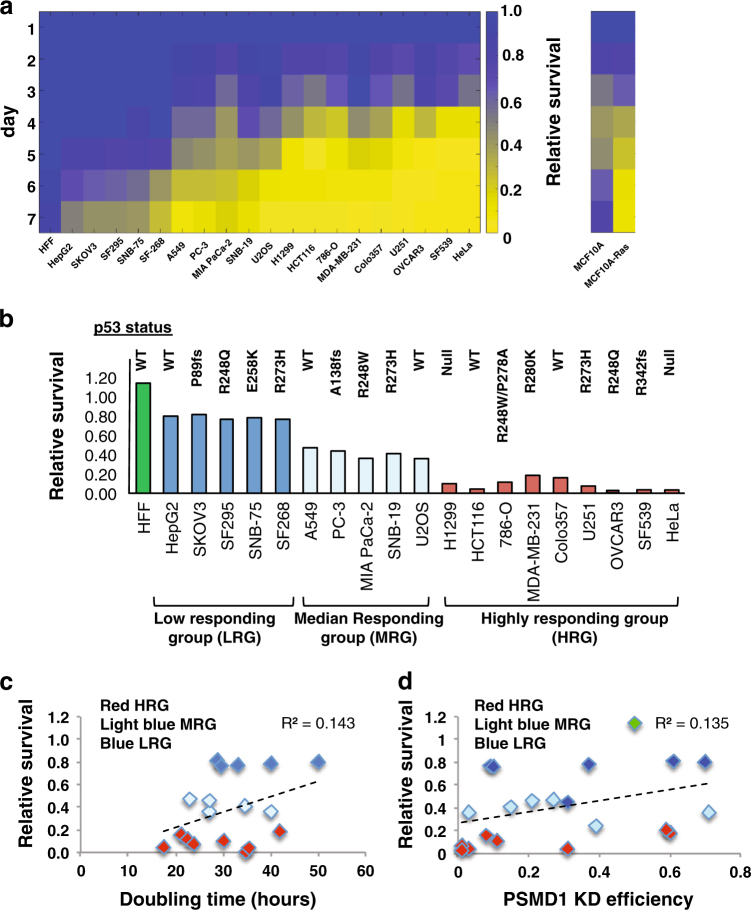
Aggressive and drug-resistant tumor cell lines are more susceptible to 26S depletion. A panel of various cell lines harboring doxycycline-inducible PSMD1 shRNA were treated with doxycycline to induce PSMD1 shRNA expression or left untreated. Alternatively, cells were transfected with siRNA against PSMD1 or luciferase gene. Cell proliferation was quantified as described in Fig. 3e. **a** Heat map analysis showing the relative survival of multiple cell lines in the course of 26S depletion (left). Heat map analysis showing relative survival of an isogenic cell line pair (MCF10A versus Ras-transformed counterpart) (right). Relative survival is calculated as a ratio of viability of PSMD1 shRNA-expressing cells to the viability of control cells measured at the same time point. **b** Relative survival of different cell lines measured at day 5 of induction. Mutational status of the p53 gene is depicted for each cell line. **c** Relative survival at day 5 plotted against the doubling time of the tested cell lines. **d** Relative survival at day 5 plotted against PSMD1 KD efficiency in the tested cell lines (PSMD1 KD efficiency is calculated as a ratio of remaining PSMD1 levels in shRNA-induced cells divided by the PSMD1 levels of control cells at the same time point)

To further generalize our findings on the 26S proteasome oncogenic dependence, we examined the effect of 26S depletion on cell viability in over 20 different cancer lines from distinct lineages and normal cells. In all tested cells the PSMD1 subunit suppression was successful (Supplementary Figure S5a–b). In all examined cancer cell lines the depletion of 26S proteasomes resulted in partial or full proliferation arrest (Fig. 5a and Supplementary Figure S5c–d). However, the normal HFF cells remained viable under this condition despite the fact that the level of depleted PSMD1 was lower than that of MDA-MB-231 cells (Supplementary Figure S5b). We compared the immortalized MCF10A to its Ras-transformed counterpart, to demonstrate the power of our analysis, highlighting the effect of transformation.

The cell lines were further stratified into four groups (non-, low, medium, and high responding) based on their response to 26S proteasome depletion (Fig. 5b). The normal non-responding group includes the HFF cells, whereas the high responding group includes U251, OVCAR3, and MDA-MB-231 that are all drug resistant, and Colo357 and H1299 that were derived from metastatic sites (ATCC). Like MDA-MB-231, Colo321, and HCT116 were also vulnerable to 26S depletion achieved by PSMD6 and PSMD11 suppression (Supplementary Figure S5e). Interestingly, there is no correlation between the status of the p53 gene and the level of viability in response to 26S depletion (Fig. 5b). These data suggest that the more aggressive the cancer lines are the more vulnerable they are to specific 26S proteasome depletion mediated by knocking down of the PSMD 19S particle subunits.

### Sensitivity to 26S proteasome depletion is not correlated with cell doubling time or depletion efficiency

The selective killing of the tumor cells upon 26S depletion could be the result of the unique cell cycle doubling time of each of the lines. Using the reported doubling time (http://www.nexcelom.com/Applications/bright-field-analysis-of-nci-60-cancer-cell-lines.php) of the examined cell lines, we plotted the survival ratio against the doubling time and found no correlation (Fig. 5c). Furthermore, the levels of PSMD1 depletion achieved in the different cell lines are variable (Supplementary Figure S5a). To explore if the observed altered dependencies of cancer cells on high 26S proteasome is correlated to the PSMD1 depletion efficiency, we plotted the degree of PSMD1 suppression versus the induced effect on cell viability and found no correlation (Fig. 5d). Thus, the increased dependency of specific cancer cells to high 26S proteasome levels is an inherent property of the specific cancer cell line.

### UPR activation in 26S-depleted cells

**Fig. 6:**
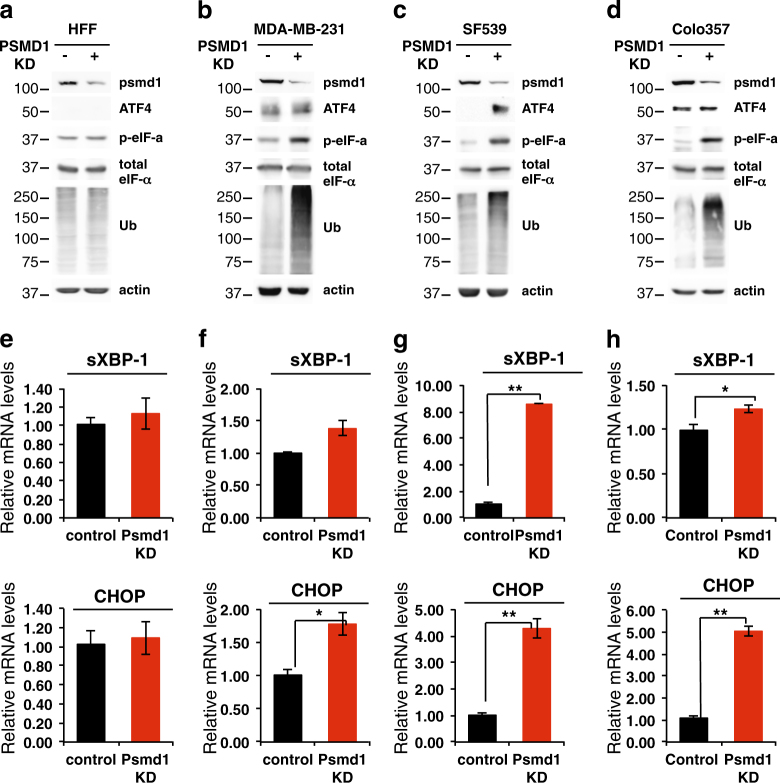
UPR activation in 26S-depleted cells. Normal fibroblasts (HFF) (**a**, **e**) and representative cell lines from the “highly responding group” MDA-MB-231 (**b**, **f**), SF539 (**c**, **g**), and Colo357 (**d**, **h**) harboring doxycycline-inducible PSMD1 shRNA were either doxycycline treated to induce PSMD1 shRNA expression or left untreated. The levels of UPR markers ATF4 and p-eIFα were analyzed by immunoblot, and of CHOP and the spliced XBP-1 (sXBP-1) mRNA were measured by qPCR. *p < 0.05, **p < 0.01

Focusing on specific cell lines from the high responding group, we further investigated the role of activation of the unfolded protein response (UPR) in mediating the 26S proteasome depletion-induced cell death. We found that 26S depletion did not increase the level of ubiquitinated proteins in HFF cells (Fig. 6a), suggesting that normal cells have excess amounts of the 26S proteasome. In contrast, in the cancer cells a marked accumulation of the ubiquitinated proteins was observed (Fig. 6b–d). This accumulation is expected to induce UPR. ATF4, a well-known hallmark of UPR activation, is highly expressed in the cancer cells. Phosphorylated eIF-α, the upstream ATF4 regulator, was only increased in the cancer cell lines. sXBP-1 level was significantly induced only in the SF539 cells upon 26S depletion (Fig. 6e–h). The level of CHOP mRNA, the downstream target of UPR, was increased in all three tested cancer cell lines. UPR also activates the IRE1–JNK pathway^42^. As expected, JNK was activated but its inhibition only marginally improved cell viability (Supplementary Figure S6a–b). Overexpression of Bcl-2 did not restore viability upon 26S depletion (Supplementary Figure S6c–d). Thus, the activation of the UPR by 26S proteasome depletion is not the major effector in inducing massive cell death.

### 26S depletion induces cytosolic condensation and nuclear distortion

**Fig. 7:**
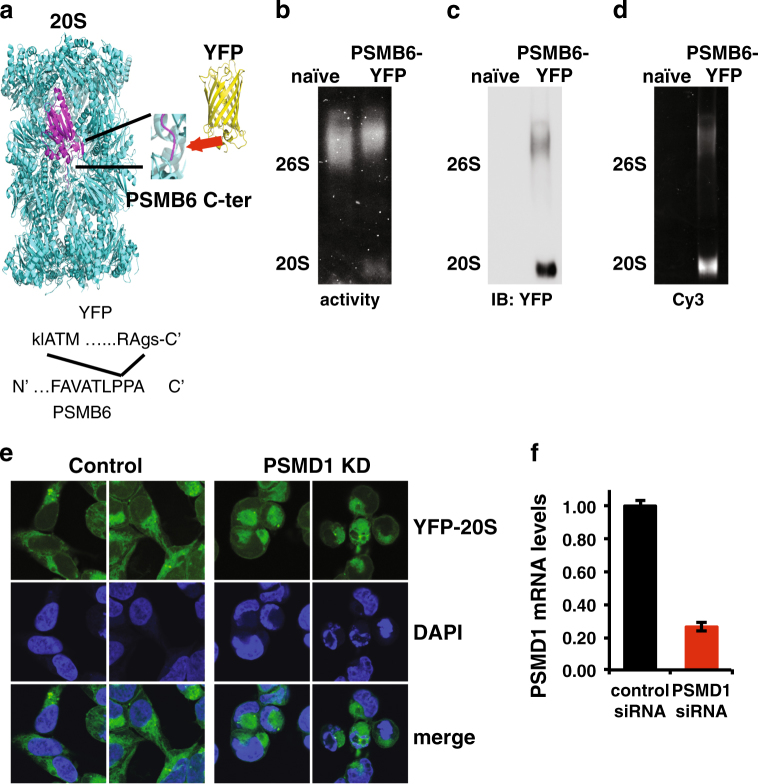
26S depletion induces cytosolic condensation and nuclear distortion. **a** Experimental strategy of CRISPR/Cas9 editing to tag the endogenous 20S subunit PSMB6 with YFP at its C terminus (the N and C terminus of the YFP sequence are shown by capital letters). **b** The 26S and 20S proteasomal complex activity of the PSMB6-YFP proteasomes was analyzed by native gel electrophoresis in both naive and PSMB6-YFP cells as described. The 20S complex is shifted a bit higher with the addition of YFP. **c** The level of the YFP proteasome complexes was examined by immunoblotting with anti-YFP or by YFP fluorescence (Cy3 filter) (**d**). **e** Cellular morphology of PSMB6-YFP 293 cells upon 26S depletion. Endogenous proteasomes are visualized by YFP, and nuclei are stained by DAPI. HEK293 PSMB6-YFP cells were transfected with siRNA targeting PSMD1 or with control siRNA targeting luciferase. PSMD1 levels in siRNA-transfected PSMB6-YFP HEK293 cells were measured by qPCR (**f**)

To visualize the effect of the 26S depletion on cellular proteasome localization at a single cell resolution, we utilized CRISPR/Cas9 editing to tag the endogenous 20S proteasome complexes with YFP (Fig. 7a–d and Supplementary Figure S7). This did not affect the ability of 26S depletion to induce cell death (Fig. 7e, f). Overall, the 26S proteasomes were localized to the cytoplasm and 26S depletion did not alter this cytoplasmic localization. However, 26S-depleted cells exhibited a rounded morphology with condensed cytoplasm and nuclear deformation and fragmentation (Fig. 7e). This nuclear morphology is often observed in cells undergoing apoptosis^43^. These data suggest that nuclear fragmentation is a likely mechanism of cell death in 26S-depleted cell lines.

### The role of caspases in 26S depletion-mediated TNBC cell death

**Fig. 8:**
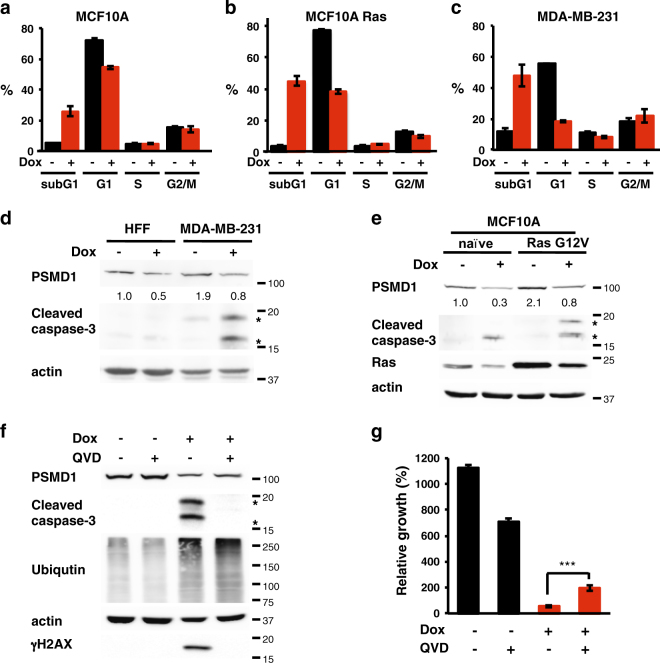
The role of caspases in 26S depletion-mediated TNBC cell death. Differential cell death response of **(a)** naive MCF10A, (**b)** Ras-transformed MCF10A, and (**c)** cancer cell line MDA-MB-231 upon 26S depletion. Cells harboring doxycycline-inducible PSMD1 shRNA were either doxycycline treated to induce PSMD1 shRNA expression or left untreated. Cell cycle was analyzed by FACS after 4 days, and cell death level was quantified by measuring subG1 fraction. **d** Caspase-3 cleavage in MDA-MB-231 cells versus normal fibroblasts was analyzed by immunoblot after 4 days of PSMD1 shRNA induction. Asterisks mark the 17 and 19 kD caspase-3 cleavage products. **e** Caspase-3 cleavage in MCF10A Ras-transformed versus naive MCF10A cells was analyzed by immunoblot after 4 days of PSMD1 shRNA induction. Role of caspases in cell death mediated by 26S depletion was examined by using pan-caspases inhibitor QVD. Cells were induced to express PSMD1 shRNA by doxycycline in the presence or absence of 25 μMQVD, and protein expression (**f**) and viability (**g**) were analyzed after 4 days

To further determine the type of cell death that is induced upon 26S proteasome depletion, we conducted Annexin V/PI double-staining analysis. The obtained data clearly suggest that the 26S-depleted cancer cells undergo apoptosis/necrosis (Supplementary Figure S8). Cells undergoing apoptosis via nuclear fragmentation can be detected by measuring the subG1 fraction of the cell cycle. Unlike the naive MCF10A cells, the Ras-transformed counterpart become enriched in subG1 fraction upon 26S depletion (Fig. 8a, b). This is the case also with the MDA-MB-231 TNBC cell line (Fig. 8c). Nuclear condensation and fragmentation is expected to induce caspase-3 cleavage. Our data confirmed this prediction and showed that caspase-3 is selectively activated in the TNBC cell line and in Ras transformed MCF10A cells (Fig. 8d, e). Upon 26S proteasome depletion the pan-caspase inhibitor, QVD, inhibited caspase cleavage/activation but unexpectedly it also diminished γH2AX activation (Fig. 8f). Under this condition, the level of the accumulated polyubiquitinated proteins in the 26S-depleted cells was not affected (Fig. 8f). QVD treatment could only achieve a partial but significant effect in preventing cell death following 26S depletion (Fig. 8g). Thus, 26S depletion-induced cancer cell death is only partially mediated by the UPR and caspase-mediated cell death.

## Discussion

We report here that transformation by H-Ras G12V of immortalized cell lines is associated with a marked increase in the level of the 26S proteasome complexes. During the process of transformation there is increased dependency on proteasome function as part of global increased burden on the protein homeostasis machinery. Genetic screen analysis in several models, including Ras transformation and triple negative breast cancer cells, revealed a strong dependency on the proteasome function^38–40^. Our analysis revealed that Ras-transformed cells cannot survive with the amount of the 26S proteasome that the naive MCF10A cells normally have. Thus, the elevated 26S proteasome level is in fact crucial for the transformed cells to survive.

Reducing the expression of one of the key 19S subunits (with shRNA or siRNA) results in reduction of 26S proteasome levels and activity and effective inhibition of the ubiquitin-dependent degradation process, while leaving the 20S catalytic particle intact and active^9^. Utilizing this experimental strategy, we characterized the dependency of cancer cells on specific 26S proteasome function. This is not only the case with the Ras-transformed cells, but holds across a panel of cancer cell lines from distinct lineages. The 19S subunits are essential^44^, therefore, complete suppression would result in cell death. However, our findings reveal differential effects of partial 19S subunit suppression. Under normal non-oncogenic conditions, cells can tolerate moderate inhibition of the 26S proteasome. Upon oncogenic transformation, despite having more 26S proteasomes, cells become increasingly more sensitive to suppression of the 26S proteasome. This is in particular the case with the drug-resistant and more aggressive tumor cell lines. Thus, oncogenic transformation entails addiction to 26S proteasome levels and function.

Targeting the proteasomes for cancer therapy proved to be effective mostly in multiple myeloma. Targeting the functional subunits of the 19S regulatory particle could serve as an attractive alternative approach with minor effects on the normal cells. Along this line, previous attempts were made to inhibit the substrate recognition and deubiquitination activity of the 19S particle components. RPN13 (ADRM1) is one of two major ubiquitin receptors within the 19S regulatory particle. The small molecule RA190 covalently binds to RPN13 and inhibits its function and is active against cervical and ovarian cancer^45^. The 19S particle further contains RPN11 (PSMD14), and two cysteine proteases USP14 and UCHL5 (UCH37) that have deubiquitinating (DUB) activity. DUB inhibitors were shown to inhibit tumor progression in certain solid tumor models^46,47^. Our findings that the transformed cells accumulate high amounts of the 26S proteasome and that depletion of a single 19S particle subunit reduces the 26S proteasome level, set the stage for a new strategy of cancer therapy. This strategy does not deal with the 19S particle-associated activities but rather depletes the 26S complex.

A critical question is why the tumor cells are addicted to high 26S proteasome levels, a behavior that is positively correlated with aggressiveness of the tested cell lines. A likely speculation is that it is due to increased protein homeostasis burden, proteotoxic stress, that is associated with cancer transformation^39,48,49^. A number of mechanisms have been proposed to explain the cytotoxic effects of proteasome inhibitors. These include NFκB inhibition^50^, expression of Myc^51^, or accumulation of the proapoptotic proteins (Bim, Bik, and Noxa)^51,52^. However, it is rather likely that these processes are the result of the activation of the unfolded protein response (UPR)^53^. In our tested cell lines, UPR and downstream JNK were activated upon 26S proteasome depletion but no strong correlation between the level of UPR activation and cell viability was observed. 26S proteasome-depleted cells show accumulation of subG1-fragmented cells and nuclear deformation, which are the hallmark of caspase activation and DNA fragmentation. However, the pan-caspase inhibitor only partially rescued cell death. Overall, our findings suggest that 26S depletion induces apoptotic cell death; however, the precise mediators of this process still need to be better characterized.

The 26S proteasome is the end point of the ubiquitin-proteasome pathway that is chiefly required for cell cycle progression. It was therefore postulated that the proliferation of cancer cells make them susceptible to proteasome inhibition. Our analysis revealed a lack of correlation between the 26S proteasome dependency and rate of cell proliferation. Moreover, in an isogenic model of Ras-transformed cells, where no significant increase in the rate of proliferation was detected, a strong increase in 26S proteasome levels and dependency was observed suggesting other mechanism(s) may be in play. This brings forward the possibility that cancer cells may exhibit altered dependencies on the 26S proteasome function that are not associated with cell cycle progression.

A model that can be suggested to explain our findings is a 26S proteasome buffer model. This model shows strong resemblance to the buffer model described for the essential chaperone Hsp90. The levels of Hsp90 in the cell are constitutively higher than required to fulfill its normal function and by that creating a reservoir buffer^54^. However, once protein homeostasis is perturbed by environmental or genetic insults, the need and dependency on Hsp90 is increased due to proteotoxic stress, diminishing the reservoir buffer of Hsp90^55–57^. In an analogous manner, our results support the model that normal cells have a reservoir of 26S proteasomes in the cell. Under normal conditions, the reduction of 26S proteasome levels is buffered by the reservoir enabling the cells to survive. However, upon transition to an oncogenic state, the 26S proteasome buffer diminishes resulting in an increased dependency on the levels of the 26S proteasome. By sampling different cancer cells, we show here that this dependency varies between the different cells. This altered dependency between different cancer cells is not correlated with the degree of 26S proteasome suppression, suggesting that the inherent cell state dictates the vulnerability to 26S proteasome depletion. Moreover, in some cancer cells and tumors there is naturally occurring (epi)genetic reduction in expression of one of the 19S subunits, suggesting that in many cancer cells and primary tumors mild reduction in the 26S proteasome buffer can be tolerated^26,58^. Thus, in the context of oncogenic transformation or in the case of more aggressive cancer cell lines where the protein homeostasis burden is expected to be increased, the dependency on 26S proteasome would be augmented as the 26S buffer would be depleted, increasing the vulnerability to targeted 26S proteasome depletion.

## Materials and methods

### Cells

MCF10A cells were cultured in DMEM: F12 medium with 5% horse serum (Gibco), 2 mM glutamine, 20 ng/ml epidermal growth factor, 10 μg/ml insulin, 0.5 mg/ml hydrocortisone, 100 ng/ml cholera toxin, 100 units/ml penicillin, and 100 μg/ml streptomycin. OVCAR3, SKOV-3, MDA-MB-231, H1299, A549, 786-O, U251, SF268, SF295, SF539, SNB-75, SNB-19, and Colo357 cells were cultured in RPMI-1640 medium (Gibco) with 10% FCS 2 mM glutamine and the antibiotics as above. HEK 293T, NIH3T3, HCT116, U2OS, HepG2, PC-3, HEK293, HeLa, and MiaPaCa-2 were cultured in DMEM and 8% FCS with antibiotics as mentioned above. Human foreskin fibroblasts (HFF) were cultured in DMEM with 10% FCS, 2 mM glutamine, and the antibiotics as above. All cells were maintained at 37 °C in a humidified incubator with 5.6% CO_2_.

### Plasmids and viral production

A lentiviral Tet-inducible TRIPZ vector with shRNAmir against 26S proteasome subunit PSMD1 was purchased from Open Biosystems (Thermo Scientific) and used to downregulate 26S levels. To downregulate PSMD6 and PSMD11 26S proteasome subunits, shRNA targeting the sequence 5′-CAGGAACTGTCCAGGTTTATT-3′ (for PSMD6) or 5′-GGACATGCAGTCGGGTATTAT-3′ (for PSMD11) were cloned into the Tet-pLKO-puro-inducible vector (Addgene plasmid #21915). To express Bcl-2 in MDA-MB-231 cells, Flag-tagged Bcl-2 was cloned into pLenti6 vector by Gateway recombination (Invitrogen). Transducing lentiviral particles were produced in HEK293T cells according to the manufacturer’s protocol. For H-Ras transformation of NIH3T3 cells, production of retrovirus particles was performed in HEK 293T cells with either pBabe empty or pBabe H-Ras G12V and psi helper plasmid. For H-Ras transformation of MCF10A cells, production of retrovirus particles was done in the Phoenix packaging cell line (kindly provided by Dr. Gary Nolan, Stanford University, Stanford, CA) with either pBabe H-Ras V12 vector or an empty pBabe vector.

### Induction of PSMD knockdown and reduction in the 26S proteasomal complexes

Cells of interest were infected with the lentivirus particles (infection conditions vary between cell lines). Selection with puromycin was employed for a week (the concentration has to be determined based on the cell type). To induce shRNA expression for knockdown of a specific PSMD subunit, cells were treated with 1 μg/ml doxycycline. Alternatively, cells were transfected with siRNAs designed against PSMD1, 6, or 11 or the luciferase gene (Dharmacon). The efficiency of the reduction in the 26S proteasomal complex was analyzed by subjecting the cellular extract to nondenaturing PAGE as previously described^59^. This enables visualization of the reduction in the 26S proteasomal complex and not only the subunit expression on the protein level (as examined by the SDS-PAGE).

### Flow cytometry

Cells were seeded at a density of 5 × 10^5^ per 9 cm dish and PSMD1 knockdown was induced by the addition of doxycycline (1 μg/ml) in the presence of puromycin (2 μg/ml). After 96 h floating and attached cells were collected and combined together, washed twice with PBS and fixed in 70% ethanol. The cells were further washed with PBS and re-suspended in 50 μg/ml RNase A and 25 μg/ml propidium iodide in PBS. For analysis of Annexin V/PI double staining, we used the Annexin V-FITC Apoptosis Detection Kit (eBioscience, San Diego, CA, USA) on unfixed cells prepared according to the manufacturer’s instructions. For each measurement, triplicates of 30,000–50,000 cells were collected by the BD LSRII flow cytometer (Becton Dickinson, Mountain View, CA, USA) and analyzed with the BD FACSDiva software (BD Biosciences).

### Cell proliferation

Cells (1–2 × 10^3^) were seeded in a well of 96-well plate in the presence of puromycin (2 μg/ml) with or without doxycycline (1 μg/ml). Cell proliferation was analyzed using the XTT assay (Biological Industries) and spectrophotometrically quantified. Relative cell growth was determined by taking the XTT result of the seeded cells as 100%. The results obtained by the XTT assay were confirmed by direct counting of cells over the period of PSMD1 KD induction. When indicated, a pan-caspase inhibitor QVD (BioVision) or JNK inhibitor SP600125 (Enzo) was added to the growth medium the next day following doxycycline induction.

In the recovery experiment, MDA-MB-231 cells (2 × 10^3^) were seeded in a well of 96-well plate in the presence of puromycin (2 μg/ml) and doxycycline (1 μg/ml). After 72 h, the cells were washed twice with the culture medium and supplemented with fresh medium with puromycin but without doxycycline for an additional 7 days. The ability of cells to recover from PSMD1 KD was compared to growth characteristics of cells that continued to be supplemented with 1 μg/ml doxycycline after the washing step or were not induced at all.

### Anchorage-independent growth

Cells (3 × 10^4^) were added to 0.5 ml of growth medium with 0.3% low-melting point agar (Bio-Rad) and layered onto 0.5 ml of 0.5% agar beds in 12-well plates in the presence of puromycin (2 μg/ml) with or without doxycycline (1 μg/ml). Colonies were photographed after 1 week.

### Colony formation assay

Cells were seeded at a density of 30 cells/cm^2^ and cultured in the presence of puromycin (2 μg/ml) with or without doxycycline (1 μg/ml) for 14 days. Colonies were fixed with 70% isopropanol for 10 min followed by staining with 0.1% crystal violet.

### Protein extraction and immunoblot analysis

Cells were collected and lysed in RIPA buffer (50 mM Tris-HCl pH 8, 150 mM NaCl, 1% Nonidet P-40 (v/v), 0.5% deoxycholate (v/v), 0.1% SDS (v/v), 1 mM DTT, and Sigma protease inhibitor mixture (P8340)) as previously described^60^, the extract was subjected to ultracentrifugation (13,000 × *g*, 15 min) and the supernatant was used as the protein extract. For detection of H2AX phosphorylation, whole cell lysates were prepared by incubation in RIPA buffer at 4 °C for 20 min, followed by sonication. Protein concentrations were determined by Bradford assay (Bio-Rad) and samples were mixed with Laemmli sample buffer [final concentration 2% SDS, 10% glycerol, 5% 2-mercaptoethanol, and 0.0625 M Tris-HCl pH 6.8], boiled for 5 min and loaded on a polyacrylamide-SDS gel. Following electrophoresis, proteins were transferred to cellulose nitrate 0.45 μm membranes (Schleicher & Schuell). The primary antibodies used are cited in Supplementary Table 1. Secondary antibodies were HRP-linked goat anti-mouse, goat anti-rabbit, and donkey anti-goat antibodies (Jackson ImmunoResearch). Signals were developed using the Ez-ECL kit (Biological Industries) and detected by ImageQuant LAS 4000 (GE).

### Nondenaturing PAGE

Proteasomal samples were loaded on a nondenaturing 4% polyacrylamide gel using the protocol previously described^59,61^. Gels were either overlaid with Suc-LLVY-AMC (50 μM) for assessment of proteasomal activity by ImageQuant LAS 4000 (GE) or transferred to nitrocellulose membranes where immunoblotting with anti-PSMA4 antibody was conducted.

### RNA interference and microscopic studies

PSMB6-YFP HEK293 cells were transfected with 100 nM of RNAi targeting PSMD1 (directed against sequence 5′-ctcatattgggaatgctta-3′) or control RNAi targeting luciferase, by using Dharmafect 4 reagent of Dharmacon. On the next day, the transfected cells were replated on glass coverslips precoated with poly-lysine. Forty eight hours following transfection, cells were fixed in 4% paraformaldehyde for 30 min and mounted in Aqua-PolyMount (Polysciences). Microscopic images were acquired using a Zeiss LSM 710 confocal scanning system, using ×63 NA (numerical aperture) 1.4 objectives, and processed using Adobe Photoshop CS6.

### RNA extraction and analysis

Total RNA was extracted using TRI Reagent (MRC). First-strand synthesis was performed using iScript cDNA synthesis kit (Quanta). qRT-PCR was performed using the LightCycler480 (Roche), with PerfeCta^®^SYBR Green FastMix mix (Quanta). All qPCRs were normalized to TBP1 mRNA levels.

### Labeling of endogenous PSMB6 by YFP

For CRISPR/Cas9-mediated modification of endogenous proteasome subunit PSMB6, HEK293 cells were transfected with pX330-U6-Chimeric_BB-CBh-hSpCas9 (gift from Feng Zhang, Addgene plasmid # 42230) encoding sgRNA targeting the stop codon of PSMB6 (TAGAATCCCAGGATTCAGGC), and a donor plasmid with 1 kb homology arms flanking an SYFP insert. The resulting construct has SYFP fused to the C terminus of PSMB6, with a four amino acid linker. The SYFP sequence was subcloned from pSYFP-C1 (gift from Dorus Gadella, Addgene plasmid #22878) using PCR.

## Electronic supplementary material

Supplementary FigS1

Supplementary FigS2

Supplementary FigS4

Supplementary FigS5a,b

Supplementary FigS5c

Supplementary FigS5d

Supplementary FigS5e

Supplementary FigS6a,b

Supplementary FigS6c,d

Supplementary FigS7

Supplementary FigS8

Supplementary figure legends

Supplementary Table 1
