# Supplementary material for: Oncogenic addiction to high 26S proteasome level
Source: Cell Death Dis. 2018 Jul 10;9(7):773. doi: 10.1038/s41419-018-0806-4 (PMC6039477; doi:10.1038/s41419-018-0806-4)
Supplement: Supplementary file 12 — Supplementary figure legends [file 41419_2018_806_MOESM12_ESM.docx]

**Supplementary Figure 1. The rate of proliferation of Ras G12V-transformed MCF10A and control cells.**

MCF10A cells devoid of pTRIPZ PSMD1 shRNA but harboring H-Ras G12V or empty vector were grown as above. Cell proliferation was estimated by XTT (a) and by direct counting (b).

**Supplementary Figure 2: Expression of 20S proteasomal subunits in Ras G12V-transformed MCF10A and control cells.**

mRNA analysis of PSMA1-7 and PSMB1-7 proteasomal subunits expression was done by qPCR as described in Figure 2.

**Supplementary Figure 4.**

**Quantification of shRNA- siRNA-mediated knockdown of 19S subunits PSMD1, PSMD6, and PSMD11.** mRNA levels of PSMD1, PSMD6 and PSMD11 during 26S depletion were quantified by qPCR. **(a)** MDA-MB-231 cells harboring doxycycline-inducible PSMD1, 6, 11 shRNA or control luciferase-targeting shRNA were induced with doxycycline to express the respective shRNA for 3 days. **(b)** MDA-MB-231 cells were transfected with siRNA targeting PSMD 1, 6 and 11 or control luciferase-targeting siRNA. mRNA was analyzed after three days by qPCR.

**Supplementary Figure 5A and B.**

**Reduction in PSMD1 levels during 26S depletion in various cell lines used in the study.** A. Protein levels were analyzed by immunoblot, and mRNA levels were measured by qPCR after 3-4 days of 26S depletion. B. The level of PSMD1 before and after depletion was quantified in HFF and MDA-MB-231 cells and compared. N=3.

**Supplementary Figure 5C**

**Nuclei deformations are observable in cancer cells (HCT116) but not in normal cells (HFF) with PSMD1 KD.** Colon carcinoma (HCT116) and normal fibroblasts (HFF) were transduced using lentiviral PSMD1 shRNA expression vector. Cells were induced to express PSMD1 shRNA by doxycycline (1μg/ml). After 4 days of induction, nuclei were stained with Hoechst 33342 and images were taken using an inverted fluorescent microscope. Expression of RFP as a marker for shRNA induction and nuclear staining by Hoechst are shown.

**Supplementary Figure 5D.**

**Relative cell growth of cell lines with and without PSMD1 knockdown.** Growth of cell lines used in the study was measured as described in Figure 3e. Red, with, and black, without doxycycline induction of shRNA.

**Supplementary Figure 5E.**

**Vulnerability of Colo321 and HCT116 upon suppression of either PSMD1, PSMD6 or PSMD11 shRNA.** Colo321 and HCT116 were transduced using lentiviral vectors for doxycycline inducible or constitutive expression of PSMD1, PSMD6, PSMD11 or control non-targeting shRNA. When appropriate, cells were induced to express shRNA by doxycycline (1μg/ml). Cell growth was analyzed by XTT, and the extent of knock down was tested by qPCR.

**Supplementary Figure 6**

**Involvement of JNK and Bcl2 in cell death mediated by 26S depletion.**

Involvement of JNK in cell death mediated by 26S depletion was examined by using JNK inhibitor SP600125 (JNKi). MDA-MB-231 cells were induced to express PSMD1 shRNA by doxycycline in the presence or absence of 5 μM JNKi, and protein expression (a) and viability (b) were analyzed. The same experimental setting was used to investigate the effect of Bcl2 overexpression. Cells harboring doxycycline-inducible PSMD1 shRNA were lentiviral transduced with Flag-Bcl-2 or empty vector. Cells were induced to express PSMD1 shRNA by doxycycline, and viability (c) and protein expression (d) were analyzed after 6 days.

**Supplementary Figure 7**

**Expression of PSMB6-YFP in HEK293 cells**

Equal amounts of HEK293 naïve and PSMB6-YFP cells lysate were analyzed for proteasomal content and activity. **(a)** Loading equivalence and transfer efficiency monitored by Ponceau S staining. **(b)** Expression of YFP, actin and the proteasomal subunit PSMA4 was quantified by immunoblot (IB).

**Supplementary Figure 8.**

AnnexinV/PI apoptosis assay for cells undergoing 26S depletion

MDA-MB-231 (a) and HeLa (b) cells were induced to express PSMD1 shRNA by doxycycline. After 4 days of induction, double staining by AnnexinV/PI was analyzed by flow cytometry. Q3: Annexin-/PI- (live cells), Q4: AnnexinV+/PI- (early apoptosis), Q2: AnnexinV+/PI+ (late apoptosis). The percentage of the overall population in each quadrant is given as a mean of 3 independent experiments with 30,000 cells. The major effect of 26S KD is observed within the late apoptotic fraction (shown on the right).
