# Supplementary material for: Oncogenic addiction to high 26S proteasome level
Source: Cell Death Dis. 2018 Jul 10;9(7):773. doi: 10.1038/s41419-018-0806-4 (PMC6039477; doi:10.1038/s41419-018-0806-4)
Supplement: Supplementary file 13 — Supplementary Table 1 [file 41419_2018_806_MOESM13_ESM.docx]

Supplementary Table I: Antibodies used 1. Primary antibodies used in the study

| Primary antibody | Source | Organism | Cat # |
| --- | --- | --- | --- |
| PSMD1 | Sigma | rabbit | S1324 |
| PSMA1 | Prof. Chaim Kahana, Weizmann Institute of Science | rabbit | - |
| PSMA4 | Prof. Chaim Kahana, Weizmann Institute of Science | rabbit | - |
| PSMD2 | Thermo Scientific | rabbit | PA1-964 |
| PSMC1 | Thermo Scientific | rabbit | PA1-965 |
| PSMC3 | Abnova | mouse | H00005702-B01P |
| Fibronectin | Sigma | mouse | F0916 |
| Myc | Weizmann Institute Ab Core Facility | mouse | - |
| c-Jun | Santa Cruz | rabbit |  |
| NQO1 | Santa Cruz | goat | C19, R20 |
| Hsc-70 | Prof. Irith Ginzburg, Weizmann Institute of Science | rabbit | - |
| actin | Sigma | mouse | A4700 |
| Beta-tubulin | Covance | mouse | MMS-435P |
| RNR-R2 | Santa Cruz | goat | sc-10844 |
| p53 | Prof. Moshe Oren, Weizmann Institute of Science | mouse | clone 1801 |
| p21 | Santa Cruz | Rabbit, mouse | sc-397, sc-6246 |
| ATF4 | Cell Signaling | rabbit | #11815 |
| eIF2α | Santa Cruz | rabbit | sc-11386 |
| pSer52 eIF2α | Thermo Scientific | rabbit | 44-728G |
| ubiquitin | Santa Cruz | mouse | sc-8017 |
| cleaved caspase-3 | Cell Signaling | rabbit | #9664 |
| pSer139 H2A.X | Upstate Biotechnology | mouse | 05-636 |
| pan Ras | Santa Cruz | mouse | sc-166691 |
| pThr183/Tyr185 SAPK/JNK | Cell Signaling | rabbit | #4668 |
| JNK1 | Cell Signaling | mouse | #3708 |
